# Micro- and nanoplastics in periodontitis: mechanistic pathways and clinical implications

**DOI:** 10.3389/fdmed.2026.1918044

**Published:** 2026-08-26

**Authors:** Bharathi Ganesan, Nina Shenoy

**Affiliations:** Nitte (Deemed to be University), AB Shetty Memorial Institute of Dental Sciences (ABSMIDS), Department of Periodontology, Mangalore, India

**Keywords:** microplastics, nanoplastics, oral exposure, oral microbiome, oxidative stress, periodontitis

## Abstract

Micro- and nanoplastics (MNPs) are increasingly recognized as pervasive environmental contaminants with potential relevance to oral and periodontal health. The oral cavity is continuously exposed to MNPs through water, food, air, oral-care products, and polymer-based dental materials, placing periodontal tissues at a plausible interface between environmental exposure and chronic inflammation. Emerging evidence suggests that MNPs can aggravate biologic processes central to periodontitis, including oxidative stress, mitochondrial dysfunction, inflammatory signaling, epithelial barrier disruption, dysbiosis, inflammasome activation, and imbalanced bone remodeling. MNPs may also act as colonization surfaces for biofilm development, alter microbial selection pressures, and contribute to antibiotic resistance gene enrichment, although direct evidence for an oral plastisphere remains limited. Epigenetic changes, including altered DNA methylation, histone marks, and microRNA profiles, provide an additional mechanism by which MNP exposure may sustain inflammatory priming. Current evidence remains predominantly preclinical, and contamination-controlled studies directly quantifying oral MNP burden in relation to periodontal phenotype are still lacking. This review summarizes current evidence on oral exposure pathways, mechanistic links between MNPs and periodontal breakdown, translational implications, and priorities for future research. Present data support viewing MNPs as plausible environmental modifiers of periodontitis rather than established independent causal agents.

## 1 Introduction

Global plastic production has increased continuously over recent decades, resulting in the widespread dissemination of micro- and nanoplastics (MNPs) across environmental, dietary, and biological compartments (1–3). These particles are generated through the fragmentation of larger plastics as well as from intentionally produced small-scale polymers, and they are now detectable in water, food, air, and multiple human tissues. Their pervasive presence has raised concern that chronic low-dose exposure may contribute to disease processes at vulnerable mucosal interfaces, particularly where barrier integrity and microbial homeostasis are already compromised (4–8).

The oral cavity is a major and continuous route of MNP exposure through ingestion, inhalation, and direct contact with polymer-containing oral-care and dental materials (9). Periodontal tissues are especially relevant because the gingival sulcus and junctional epithelium form a highly specialized but permeable barrier adjacent to dense subgingival biofilms. This anatomical setting makes the periodontium susceptible to environmental cofactors that may intensify oxidative stress, alter microbial ecology, and amplify inflammatory signaling (10).

Periodontitis represents a substantial global health burden, with severe forms affecting more than 1.1 billion people worldwide. As one of the most common non-communicable diseases, it carries significant public health and economic implications (11). Periodontitis is a chronic inflammatory disease driven by dysbiotic biofilms and a dysregulated host response, leading to connective tissue destruction and alveolar bone loss. Although microbial dysbiosis remains central to its pathogenesis, periodontitis is now understood as a multifactorial condition shaped by host, behavioral, and environmental influences (12, 13). In this broader framework, MNPs may represent an underexplored environmental modifier that can exacerbate periodontal breakdown through direct cellular toxicity, immune dysregulation, disruption of the epithelial barrier, and promotion of a pro-inflammatory microenvironment (14).

Emerging experimental evidence suggests that MNP exposure can increase reactive oxygen species production, impair antioxidant defenses, activate inflammasome pathways, and alter fibroblast, epithelial, and immune cell function. MNPs may also facilitate subgingival biofilm adhesion by providing surfaces for plastisphere formation and selective enrichment of periodontopathogenic taxa (15). In addition, co-adsorbed additives and associated chemicals may further magnify inflammatory and endocrine-disrupting effects (16). However, the current evidence base remains largely preclinical, and direct clinical data linking oral MNP burden with periodontal outcomes is still limited.

This review therefore addresses objectives on oral MNP exposure pathways, periodontal retention, mechanistic contributions to tissue destruction, and translational implications for periodontal research and practice. By integrating the existing experimental literature, it aims to clarify whether MNPs should be considered a meaningful environmental contributor to periodontitis and a potential target for future biomonitoring and preventive strategies.

## 2 Oral exposure pathways and periodontal retention

### 2.1 Environmental, dietary, and product-related sources

The oral cavity represents a continuous and multisource route of MNP exposure. Contaminated drinking water is among the most significant contributors, with bottled water releasing approximately 240 nanoplastic particles per liter, while estimated annual dietary ingestion reaches 39,000–52,000 particles, rising to over 100,000 when airborne deposition is included (17, 18). Oral-care products — including certain toothpastes, mouthwashes, and polytetrafluoroethylene (PTFE)-coated dental floss — introduce particles repeatedly and directly at the gingival margin, a site with inherently limited self-cleansing capacity (19, 20). The 2023 European Chemicals Agency (ECHA) restriction on intentionally added microplastics in rinse-off products marks meaningful regulatory progress, though global implementation remains incomplete (21).

### 2.2 Iatrogenic dental sources

Dental procedures constitute a clinically distinctive and underrecognized exposure route. High-speed composite preparation aerosolizes respirable polymethylmethacrylate (PMMA) and bisphenol A-glycidyl methacrylate (Bis-GMA) nanoparticles, while ultrasonic scaling may deposit particle loads directly onto instrumented periodontal tissues (22, 23). Orthodontic aligners, polymer-based impression materials, and infection-control consumables further contribute through nanoscale particle shedding under intraoral mechanical and chemical stress (24). The clinical significance of these exposures lies in their proximity to sites of active inflammation or epithelial disruption, where increased mucosal permeability may enhance local particle uptake and retention (19).

### 2.3 Periodontal compartments as retention depots

Once introduced into the oral environment, MNPs may accumulate within dental plaque, saliva, gingival crevicular fluid, subgingival calculus, and periodontal pockets. The structured plaque biofilm provides surfaces for particle adhesion, while salivary protein corona formation modifies particle surface properties and may promote biofilm–host tissue interactions (25–27). Inflamed periodontal sites are likely to favor particle persistence through increased pocket depth and reduced local clearance. Retention within subgingival calculus or junctional epithelium further shelters particles from salivary clearance, and the hydrophobic particle surface may adsorb microbial products, virulence factors, and environmental chemicals (28). This local accumulation may increase the opportunity for prolonged contact with gingival epithelial cells, fibroblasts, and immune cells, thereby creating conditions that could facilitate downstream inflammatory, microbial, and tissue-destructive effects.

## 3 Mechanistic pathways of MNP-mediated periodontal damage

MNPs may contribute to periodontal breakdown through multiple interrelated biological pathways rather than a single dominant mechanism. Current evidence, derived primarily from *in vitro* systems and limited *in vivo* models, suggests that MNP exposure can intensify oxidative stress, amplify inflammatory signaling, impair epithelial barrier integrity, dysregulate bone remodeling, disturb microbial ecology, and induce epigenetic changes relevant to chronic periodontal inflammation. While these pathways remain incompletely validated in human disease, they provide a biologically plausible framework for understanding how MNPs may modify periodontal susceptibility and progression. Table 1 summarizes key experimental studies examining the cellular effects of MNPs on periodontal and oral tissues.

**Table 1:** Evidence summary of micro-/nano-plastic effects in periodontal-related cell and tissue models.

Author, year	Study type	Plastic type & size	Cell/model	Key findings
Caputi S et al. (2022) (29)	*In vitro*	Environmental MPs, mixed sizes/types (Adriatic Sea)	Human gingival fibroblasts	NF-κB/MyD88/NLRP3 inflammatory pathway activation; increased inflammatory markers at gene and protein levels.
Wu Z et al. (2025) (30)	*In vitro* + Human calculus analysis	Polyethylene (PE) MNPs; identified as dominant component (32.7%) of dental calculus	Human gingival fibroblasts	Dose-dependent cytotoxicity; reduced cell viability and migration; apoptosis; ROS generation; NF-κB activation; IL-1β and IL-6 upregulation.
Pan C et al. (2025) (31)	*In vitro*/Animal	Polystyrene MPs (chronic oral exposure)	Bone stem cells + mouse model	Osteoporosis via disrupted osteoblast/osteoclast balance; bone stem cell senescence via NF-κB; excessive RANKL → increased osteoclastogenesis.
Di Cintio F et al. (2025) (32)	*In vitro*	Polystyrene MPs, 1 µm	Human gingival fibroblasts	Intracellular internalization (∼10% of cells); enhanced cell motility; proteomic changes indicating inflammatory activation and altered proteostasis.
Chen C et al. (2026) (33)	*In vitro*/*ex vivo*	Polystyrene nano-plastics	Macrophages + periodontitis patient saliva model	Periodontitis-specific protein corona (enriched with apolipoproteins, complement proteins) enhances macrophage uptake and exacerbates periodontal inflammation.

### 3.1 Oxidative stress, mitochondrial dysfunction, and Nrf2 suppression

Oxidative stress represents the most consistently reported and mechanistically substantiated consequence of MNP exposure in periodontal-relevant models. In gingival fibroblasts, polystyrene and polyethylene terephthalate (PET)-derived particles elevate reactive oxygen species (ROS), lipid peroxidation, and oxidative DNA damage while depleting superoxide dismutase, catalase, and glutathione peroxidase — effects that compound the redox burden already established by neutrophil hyperactivity and bacterial challenge in periodontitis (10, 32, 34).

Mitochondrial dysfunction likely operates as a critical upstream driver, with nanoplastics disrupting membrane potential, impairing adenosine triphosphate (ATP) synthesis, and amplifying mitochondrial ROS output (35). In metabolically stressed periodontal tissues, this mechanism may bridge MNP exposure to apoptosis, senescence-associated secretory signaling, and defective tissue repair (36). Compounding this, MNPs may suppress the Nrf2/ARE cytoprotective axis via enhanced Keap1-mediated ubiquitination, dismantling the principal antioxidant defense of gingival tissues and establishing a self-reinforcing cycle of redox imbalance and impaired healing (37–40).

### 3.2 NF-κB signaling and MAPK activation

Nuclear factor kappa B (NF-κB) is the master transcriptional regulator of periodontal inflammation, and its activation by MNPs has been directly demonstrated in gingival fibroblasts and epithelial cells (41). MNP-induced ROS triggers IKKβ-mediated degradation of IκBα, driving nuclear translocation of NF-κB p65 and transcription of IL-1β, IL-6, IL-8, TNF-α, cyclooxygenase-2 (COX-2), and inducible nitric oxide synthase (iNOS), a cytokine profile mechanistically homologous to that induced by dysbiotic subgingival biofilms (42, 43). Chronic MNP exposure may therefore lower the NF-κB activation threshold, rendering periodontal tissues disproportionately susceptible to inflammatory escalation in the presence of established dysbiosis. Parallel activation of mitogen-activated protein kinase (MAPK) pathways, including extracellular signal-regulated kinase 1/2 (ERK1/2), p38, and c-Jun N-terminal kinase (JNK), may further amplify this response (41), contributing to apoptosis, extracellular matrix degradation, and reduced reparative capacity in connective tissue cells. Together, these findings suggest that MNP exposure may lower the threshold for exaggerated inflammatory responses in periodontal tissues already challenged by microbial and host-derived stressors.

### 3.3 NLRP3 inflammasome activation and pyroptosis

In addition to broader inflammatory signalling, MNPs may activate the NOD-like receptor protein 3 (NLRP3) inflammasome, an important mediator of IL-1β- and IL-18-driven inflammation. Experimental studies in gingival fibroblasts indicate that polystyrene microplastics can stimulate NF-κB/MyD88-dependent NLRP3 activation, followed by caspase-1-mediated cytokine maturation and pyroptotic cell death (43, 44). This mechanism is potentially relevant to periodontitis because it may intensify local tissue damage and promote propagation of inflammation within the periodontal pocket.

Nano-plastics may also contribute to inflammasome activation through intracellular trafficking and lysosomal injury. Following endocytic uptake, lysosomal membrane destabilisation and cathepsin B release have been proposed as upstream triggers of NLRP3 activation in experimental systems (45). While the periodontal significance of this pathway remains to be confirmed *in vivo*, it represents a plausible link between particle internalisation and amplified innate immune activation.

### 3.4 Epithelial barrier disruption and immune dysregulation

The sulcular and junctional epithelium form the primary biological interface between the subgingival biofilm and the connective tissue. MNP exposure may compromise this barrier by downregulating tight junction proteins — occludin, claudin-1, and ZO-1 — through ROS-mediated mechanisms, increasing paracellular permeability to bacterial antigens and inflammatory mediators (32, 46). A proposed “Trojan horse” mechanism, whereby MNP surfaces adsorb and concentrate lipopolysaccharide (LPS) and microbial virulence factors at the epithelial interface, may further amplify innate immune activation in already-inflamed tissues. Beyond innate responses, MNP-driven elevations in IL-1β and IL-6 may skew adaptive immunity toward Th17-dominant responses while attenuating regulatory T-cell restraint — a pattern strongly associated with periodontal tissue destruction (42). Although direct evidence for MNP-induced Th17/Treg imbalance in periodontal tissues remains unavailable, this mechanism is biologically plausible and represents a priority area for future research in oral immunology.

### 3.5 RANKL/OPG dysregulation and alveolar bone loss

Alveolar bone loss is a defining feature of periodontitis, and emerging evidence implicates MNPs in disruption of the receptor activator of nuclear factor-κB ligand (RANKL)/osteoprotegerin (OPG)/RANK axis. MNP exposure has been associated with upregulation of RANKL and suppression of OPG in macrophage and osteoblast models, favoring osteoclast differentiation and bone resorption (47, 48). Crucially, *in vivo* evidence from a rat experimental periodontitis model demonstrates that oral polystyrene microplastic exposure significantly exacerbates alveolar bone loss, providing the most direct experimental evidence to date for MNP-mediated periodontal bone destruction (48, 49). These effects are compounded by MNP-driven TNF-α and IL-1β elevation, which independently amplify osteoclastogenesis, while oxidative suppression of Runx2 and Wnt/β-catenin signaling simultaneously impairs osteoblast differentiation and mineralization (50). However, the magnitude of this contribution relative to established periodontal risk factors requires quantification in longitudinal *in vivo* and human studies.

### 3.6 Oral microbiome dysbiosis and the plastisphere

Beyond direct cellular toxicity, MNPs may alter the ecology of the subgingival microbiome by integrating into dental plaque as additional colonization surfaces, creating plastisphere-like niches within the periodontal sulcus (51, 52). Experimental evidence suggests that such particle-associated communities may selectively enrich for periodontopathogenic taxa — including *Porphyromonas gingivalis, Fusobacterium nucleatum, Tannerella forsythia,* and *Treponema denticola* — while enhancing extracellular polymeric substance production and increasing biofilm recalcitrance (53, 54). However, it must be explicitly noted that no published study has directly characterized an oral or subgingival plastisphere in humans, and the majority of supporting evidence is extrapolated from marine, aquatic, and non-oral experimental systems (50, 51). Enrichment of antibiotic resistance genes via mobile genetic element transfer within plastisphere-associated communities represents an additional concern (55, 56). Co-adsorbed chemical additives — including triclosan, bisphenol A (BPA), phthalates, and per- and polyfluoroalkyl substances (PFAS) — may compound these effects by altering microbial selection pressures and compromising host immune responses, though disentangling additive-mediated from particle-mediated microbiome effects remains a key methodological challenge. Because current evidence is still indirect and often extrapolated from non-oral systems, the concept of an oral plastisphere remains promising but requires more direct periodontal validation.

### 3.7 Epigenetic reprogramming

**Figure 1:**
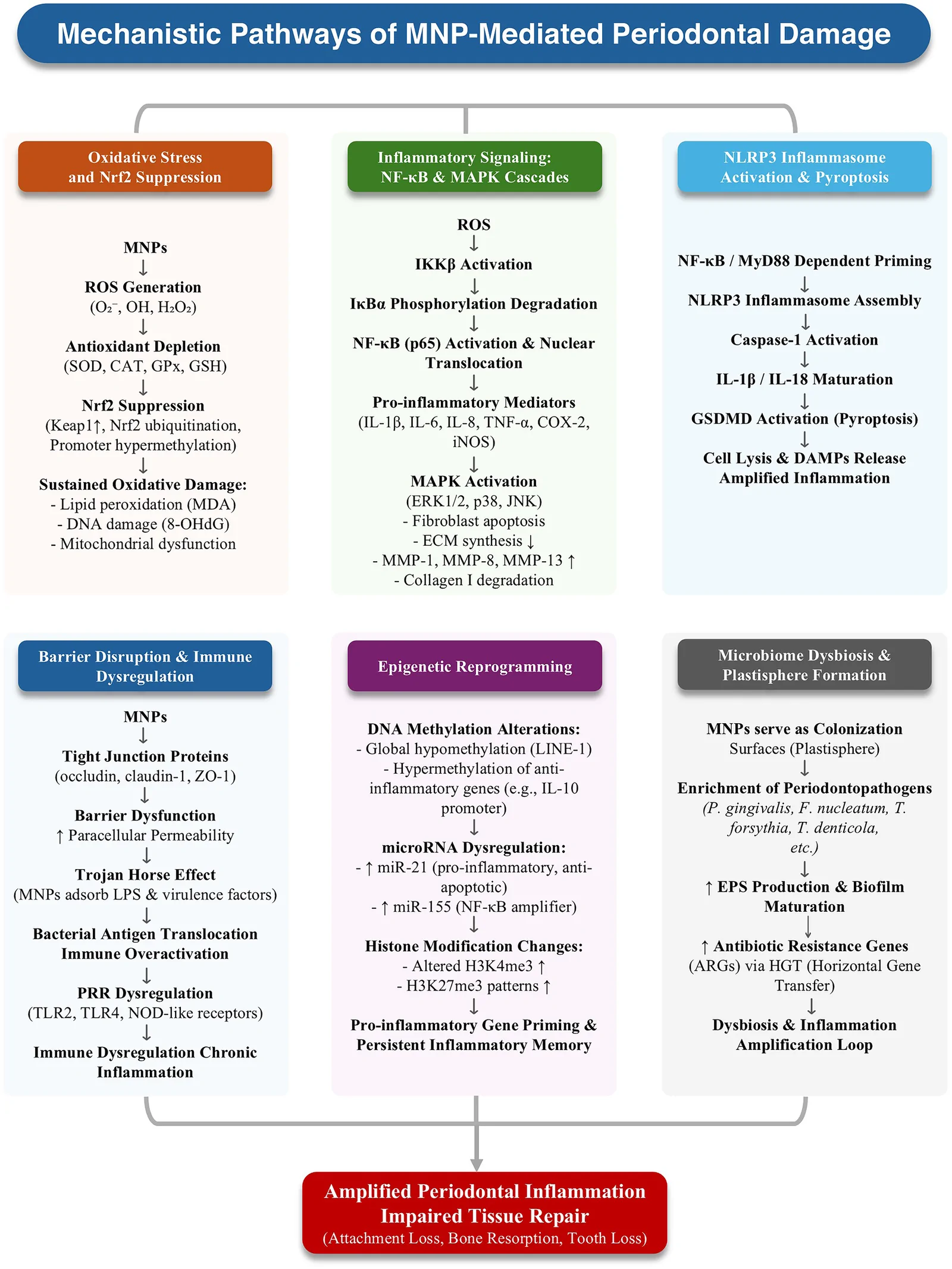
Proposed mechanistic pathways of MNP-mediated periodontal damage. This figure illustrates pathways by which micro/nanoplastics (MNPs) may contribute to periodontal breakdown, drawn from *in vitro* and animal model evidence. Pathways including oxidative stress, inflammatory signaling, NLRP3 inflammasome activation, epithelial barrier disruption, and microbiome dysbiosis are supported by experimental data from periodontal-relevant cell systems, though direct human evidence is lacking. Epigenetic reprogramming is included as a speculative pathway based on findings from non-oral cell systems, with no studies yet conducted in periodontal tissues. ARGs, antibiotic resistance genes; GSH, glutathione; MyD88, myeloid differentiation primary response protein 88; NOD, nucleotide-binding oligomerization domain.

Epigenetic modification represents an emerging mechanism through which MNP exposure may exert sustained effects on periodontal tissues. Polystyrene nanoparticle exposure has been associated with global hypomethylation of long interspersed nuclear element-1 (LINE-1) repetitive elements and locus-specific hypermethylation of anti-inflammatory gene promoters, potentially sustaining pro-inflammatory programs independently of bacterial stimulation (57, 58). MNP exposure may additionally upregulate miR-21 and miR-155 — both linked to periodontitis severity — suggesting microRNA (miRNA)-mediated amplification of NF-κB-driven inflammation. Preliminary evidence further implicates alterations in histone H3K4me3 and H3K27me3 marks at inflammatory gene loci (59, 60). Collectively, these changes raise the possibility that MNP exposure could leave periodontal cells epigenetically primed for pro-inflammatory responses long after particle clearance. However, the reversibility, tissue specificity, and clinical significance of these modifications remain unestablished and require direct periodontal validation. Figure 1 illustrates the proposed mechanistic pathways of MNP-mediated periodontal damage. Collectively, these pathways remain experimentally proposed rather than clinically validated, and their relative contributions to human periodontal disease await confirmation through dedicated translational and longitudinal studies.

## 4 Systemic dimensions: MNPs, periodontitis, and comorbidities

Periodontitis is closely linked to systemic disorders characterized by chronic inflammation and oxidative stress, including cardiovascular disease, diabetes mellitus, and adverse pregnancy outcomes. Because MNP exposure has also been associated with inflammatory, oxidative, and endocrine-disrupting effects, it is plausible that these exposures may interact with pathways already implicated in oral-systemic disease relationships (61). In this context, MNPs may represent an additional environmental modifier capable of amplifying host susceptibility rather than an independent primary cause of systemic disease.

Emerging reports of MNP detection in atherosclerotic plaques and placental tissues, together with evidence that plastic-associated additives can influence metabolic and immune regulation, support the need to consider MNP exposure within broader periodontal risk frameworks (61, 62). However, the current evidence remains largely associative and insufficient to establish causal links among oral MNP burden, periodontitis severity, and systemic clinical outcomes. Future studies should therefore integrate periodontal phenotyping with MNP biomonitoring and systemic health assessment to clarify whether these shared pathways have meaningful clinical implications.

## 5 Detection strategies and biomarker opportunities

### 5.1 Detection of MNPs in oral biospecimens

Detection of MNPs in saliva, gingival crevicular fluid (GCF), and gingival tissues remains methodologically challenging because these matrices are biologically complex and highly susceptible to external contamination (19, 20). Reliable analysis requires stringent contamination control, including the use of non-synthetic laboratory materials, filtered reagents, glassware, procedural blanks, and carefully standardized collection and processing protocols. Analytical approaches such as micro-Raman spectroscopy, micro-FTIR, and pyrolysis-GC/MS offer complementary strengths, but none is yet readily adaptable to routine periodontal practice (26, 27). A recent biomonitoring study detected MNPs in saliva and hand samples from young children, supporting the feasibility of oral biospecimen collection for MNP analysis (63), though validated, contamination-controlled protocols for periodontal research settings are still lacking.

### 5.2 Biomarker opportunities for periodontal translation

Because direct detection of MNPs in periodontal samples is not yet clinically practical, surrogate biomarker approaches may provide a more feasible translational pathway. Candidate biomarkers include oxidative stress markers such as malondialdehyde, 8-OHdG, and total antioxidant capacity; inflammatory mediators including IL-1β, IL-6, IL-8, and TNF-α; matrix-remodeling markers such as MMP-8, MMP-9, and TIMP-1; and epigenetic markers including LINE-1 methylation, miR-21, and miR-155. These markers are biologically relevant because they map onto the main pathways proposed to mediate MNP-associated periodontal injury (64–66). These map directly onto the pathways proposed to mediate MNP-associated periodontal injury. A proposed multiparametric salivary biomarker framework is outlined in Table 2. These markers should currently be regarded as exploratory; most lack specificity for MNP exposure, and their values may be influenced by established periodontal and systemic confounders. Therefore, standardized saliva collection (fasting state, polymer-free devices) and establishment of population reference intervals are prerequisites for clinical translation.

**Table 2:** Proposed multiparametric biomarker panel for MNP-associated periodontal assessment.

Biomarker category	Specific markers	Mechanistic basis	Clinical utility
Oxidative stress	MDA, 8-OHdG, TAC	ROS-driven lipid and DNA oxidation	Early indicator of subclinical periodontal stress
Pro-inflammatory cytokines	IL-1β, IL-6, IL-8, TNF-α	NF-κB, MAPK, NLRP3 activation	Tracks inflammatory phenotype; treatment response monitoring
Matrix-degrading enzymes	MMP-8, MMP-9, TIMP-1	ECM catabolism; attachment loss	Assesses disease severity and healing response
Innate immune/epithelial markers	Calprotectin, S100A8/A9, lactoferrin	Mucosal barrier stress; neutrophil/macrophage activation	Evaluates barrier integrity and immune activation
Microbiome-derived markers	*P. gingivalis*, *F. nucleatum* DNA/RNA	Plastisphere-driven dysbiosis; pathobiont enrichment	Dysbiosis indexing and risk stratification
Epigenetic markers	LINE-1 methylation, miR-21, miR-155	MNP epigenetic reprogramming	Emerging population-level exposure indicator

## 6 Clinical and translational implications

Although direct clinical evidence remains limited, the mechanistic literature suggests that MNP exposure may have implications for periodontal risk assessment, biomaterial selection, and patient counselling. These implications should currently be interpreted as hypothesis-generating rather than practice-changing, given that most supporting data derive from experimental models. Nevertheless, emerging evidence justifies considering MNP exposure as a possible modifying factor in patients with persistent inflammation, unexplained disease progression, or poor response to conventional therapy.

### 6.1 Periodontal management in potentially high-exposure patients

Conventional periodontal care, meticulous biofilm control, subgingival debridement, and management of established risk factors, including smoking and diabetes, remain the primary therapeutic approach. Where biofilm persistence or disproportionate inflammatory responses are observed, MNP exposure may be considered a potential contributing factor. In such cases, adjunctive subgingival antimicrobial irrigation (chlorhexidine 0.12%–0.2%), photodynamic therapy, or diode laser decontamination may help address structurally reinforced MNP-associated biofilms (67). Sub-antimicrobial-dose doxycycline (20 mg twice daily) represents a rationally selected host-modulatory option given its capacity to inhibit matrix metalloproteinases (MMPs) and suppress NF-κB-mediated inflammatory pathways implicated in MNP-driven tissue damage (68). These considerations supplement established therapy such as the EFP S3-level guideline for stage I–III periodontitis (69). Emerging pro-resolving lipid mediators show mechanistic promise but lack periodontal-specific clinical validation. No intervention has yet been validated specifically for MNP-associated periodontal injury; current evidence supports clinical awareness more than a distinct treatment algorithm.

### 6.2 Material selection and iatrogenic exposure reduction

Dental materials and procedures may contribute to cumulative oral MNP burden. Where clinically appropriate, mineral- or silica-based prophylaxis pastes, biodegradable membranes for guided tissue regeneration, and naturally derived drug-delivery carriers (chitosan, alginate, collagen) may be preferred over synthetic polymer-based alternatives (15, 20). Water-cooled low-speed instrumentation during composite finishing and 0.2 µm waterline filtration can reduce aerosolized particle exposure (22, 23). Patients undergoing prolonged clear aligner therapy, particularly those with a periodontitis history, warrant heightened periodontal monitoring (24). However, comparative clinical evidence is insufficient to support material recommendations based solely on MNP concerns; established criteria of safety, efficacy, and biocompatibility should remain primary determinants, with MNP exposure regarded as an emerging supplementary consideration.

### 6.3 Patient counselling and preventive relevance

Patient counselling offers a practical avenue for translating emerging evidence into preventive care. Practical measures — avoiding plastic containers during microwave heating, selecting certified microplastic-free oral care products, and preferring minimally processed foods and filtered water — may help reduce cumulative exposure (10, 21, 47). While the direct periodontal benefit of such measures remains unestablished, they align with broader preventive health frameworks and are particularly relevant for immunologically susceptible patients (14).

Overall, the clinical relevance of MNPs in periodontology currently lies in raising awareness of a potentially important environmental modifier of host–microbe interactions. As detection methods, biomarker approaches, and longitudinal human data mature, MNP exposure may become more meaningfully integrated into periodontal prevention, risk stratification, and translational research.

## 7 Research gaps and future directions

The evidence linking MNPs to periodontitis remains preliminary, with most data derived from *in vitro* systems or non-oral experimental models. Direct human evidence relating quantified oral MNP burden to periodontal phenotype, disease severity, or treatment response is lacking, leaving biological plausibility established but clinical causality unproven.

Several priorities emerge for future investigation. First, standardized, contamination-controlled protocols for collecting and analyzing oral biospecimens — saliva, gingival crevicular fluid, subgingival plaque, and gingival tissue — are essential to enable cross-study comparison and define exposure–response relationships. Second, mechanistic studies must adopt biologically relevant exposure conditions, incorporating clinically appropriate doses, polymer-specific and mixed-particle exposures, and models that reproduce the periodontal microenvironment, including biofilm dynamics, epithelial barrier function, immune signaling, and alveolar bone remodeling. Third, prospective cohort studies integrating periodontal phenotyping with oral MNP quantification and biomarker profiling are needed to determine whether MNP burden associates with disease initiation, progression, or treatment resistance, while adequately controlling for confounders such as smoking, diabetes, diet, and oral hygiene. Larger-scale biomonitoring surveillance, building on feasibility shown in paediatric salivary studies, together with pilot exposure-reduction trials, would strengthen the translational evidence base.

Finally, candidate oxidative, inflammatory, microbial, and epigenetic markers require validation in well-characterized populations to assess whether MNP-associated signatures meaningfully improve periodontal risk stratification beyond established clinical indicators.

## 8 Conclusion

MNPs are emerging environmental contaminants with increasing relevance to periodontal biology, and current evidence supports their role as plausible modifiers of periodontitis rather than established independent causal agents. Experimental findings indicate that MNP exposure may intensify periodontal tissue injury through convergent effects on oxidative stress, inflammatory signaling, epithelial barrier integrity, microbial dysbiosis, and bone remodeling, thereby creating conditions that favor disease initiation, persistence, and progression. These mechanisms are biologically credible, but the present evidence base remains dominated by *in vitro* and limited *in vivo* models, with direct human periodontal data still lacking. Accordingly, the principal challenge is no longer mechanistic plausibility but clinical translation. Progress in this field will require standardized contamination-controlled methods for oral biospecimen analysis, prospective human studies linking quantified MNP burden with periodontal phenotypes, and validation of biomarker-based approaches for exposure assessment and risk stratification. Realizing this potential will require coordinated efforts across exposure characterization, mechanistic/biomarker research, and clinical translation, supported by collaboration among periodontal researchers, environmental scientists, and regulatory bodies building on measures such as the 2023 ECHA microplastics restriction. As environmental plastic exposure continues to increase, clarifying the contribution of MNPs to periodontal disease may open an important new dimension in preventive, diagnostic, and translational periodontology.
